# Iatrogenic Left Main Bronchus Injury following Atraumatic Double Lumen Endotracheal Tube Placement

**DOI:** 10.1155/2013/524348

**Published:** 2013-10-31

**Authors:** William R. Hartman, Michael Brown, James Hannon

**Affiliations:** Department of Anesthesiology, Mayo Clinic, Rochester, MN, USA

## Abstract

Tracheobronchial disruption is an uncommon but severe complication of double lumen endotracheal tube placement. The physical properties of a double lumen tube (large external diameter and length) make tracheobronchial injury more common than that associated with smaller single lumen endotracheal tubes. Here we present the case of an iatrogenic left main bronchus injury caused by placement of a double lumen tube in an otherwise unremarkable airway.

## 1. Introduction

Thoracic surgery procedures requiring lung isolation are often performed with the assistance of a double lumen endotracheal tube (DLT). While placement of DLTs is routine and safe in experienced hands, it is not without risk. A rare complication is airway rupture, perhaps due to DLTs having a larger external diameter compared to a single lumen tube and a stiff stylette used for ease of proper endotracheal tube placement [1, 2]. Early recognition of airway rupture, evaluation of the defect, and repair of the airway are critical to optimal patient outcome [3]. 

Here we present the case of a 52-year-old woman who presented to the operating room for removal of a right upper lobe mass via right thoracotomy. Despite a seemingly atraumatic intubation with a 35-French left double lumen endotracheal tube, a significant tear in her left main bronchus was identified intraoperatively. 

## 2. Case Report

 A 158 cm, 93 kg (BMI 37.4), 52-year-old woman presented to a tertiary care center for evaluation of a Merkel cell tumor in her right forearm. Wide resection of this forearm tumor and an axillary lymph node dissection were successfully performed. During the course of her evaluation, however, a suspicious mass in her right upper lobe was identified on chest X-ray, and a 2.7 × 2.2 × 2.9 cm hypermetabolic solid nodule in the right apex was confirmed with CT imaging. The patient was asymptomatic. Preoperative pulmonary function tests were normal. Patient was deemed to be optimized for a thoracic surgical procedure and was brought to the operating room. 

 After routine intravenous sedation with Midazolam and Fentanyl, an epidural was placed at the T6-7 vertebral interspace. Patient was returned to the supine position where general anesthesia was induced intravenously with fentanyl, lidocaine, propofol, and succinylcholine. Her airway was secured via direct laryngoscopy (grade one view) and placement of a styleted 35-French left double lumen endotracheal tube without difficulty. Once the tracheal cuff had passed through the cords, the stylette was removed and bilateral breath sounds were confirmed. Proper placement of the bronchial lumen of the tube in the left main bronchus was confirmed with fiberoptic bronchoscopy. The patient was placed in lateral position for her surgical procedure, and proper placement of the DLT was once again confirmed with the fiberoptic bronchoscope. No blood or signs of bronchial or tracheal trauma were observed.

Following initiation of single lung ventilation, a surgical decision was made to proceed with a right upper lobectomy which was performed in the usual fashion. Individual branches of the pulmonary artery to the right upper lobe were identified and doubly ligated with 2-0 silk suture. Right superior pulmonary vein was mobilized. Branches going to the right upper lobe were identified and divided with silk stick ties and free ties. The fissures between the middle lobe and upper lobe were completed with multiple firings of a GIA stapler. All interlobar lymph nodes were resected. The right upper lobe bronchus was sharply dissected free. The right lung was reinflated and the bronchial stump was competent to 40 cm pressure; however, with positive pressure ventilation and the right hemithorax filled with saline, a small air leak was identified. Further evaluation revealed the air leak to be arising from a long linear tear located along the left mainstem bronchus and extending from the carina as far down the left mainstem bronchus as could be visualized. The double-lumen endotracheal tube was identified in the region of injury. This left main bronchus injury was not evident during the course of the case because the bronchial balloon had successfully isolated the left lung. The injury and resultant air leak only became apparent with deflation of the bronchial balloon and the resumption of right lung ventilation. 

Due to the location of the injury and concern that the current exposure would not provide adequate visualization for surgical repair, a right thoracotomy was performed. The bronchial lumen of the DLT was inserted distal to the defect with the use of a flexible bronchoscope and surgeon observation. With the ability to ventilate the lung distal to the airway rupture, the defect was repaired with a combination of a pericardial patch and pleural flap. No air leak was identified upon completion of the repair, and the chest was closed in the usual fashion. 

**Figure 1:**
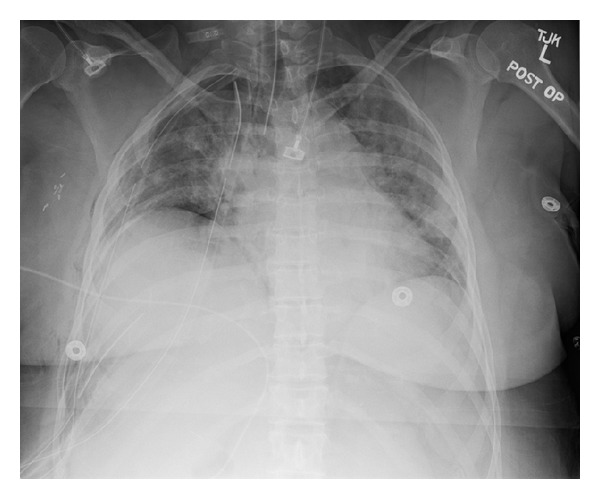
Postoperative chest X-ray. Seen are a single lumen ETT (DLT was replaced with SLT for bronchoscopy), three right chest tubes, and tiny right apical and basilar pneumothoraces. Infiltrate is observed throughout both lungs.

**Figure 2:**
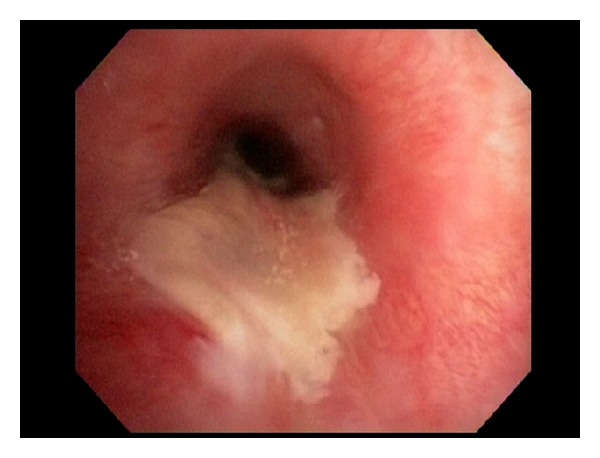
Endobronchial view of left main bronchus repair.

**Figure 3:**
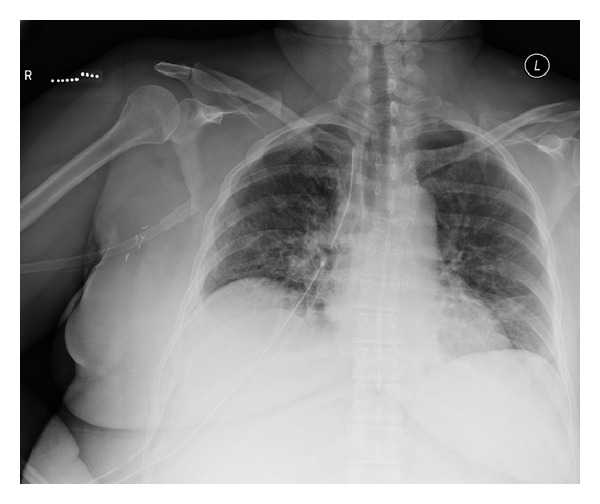
Postoperative day 7 chest X-ray. No evidence of left sided air leak or residual left main bronchus rupture.

The patient was returned to the supine position, and the anesthesiologist removed the double lumen endotracheal tube under direct vision with a video laryngoscope and placed a single lumen endotracheal tube (Figures 1 and 3). At this time, a bronchoscopist conducted a thorough examination of the airway, revealing findings consistent with a right upper lobectomy with intact surgical stump. The remainder of the visible tracheobronchial tree on the right side was normal and fully patent. Examination of the left tracheobronchial tree, findings consistent with a left mainstem bronchial injury beginning approximately 0.5 cm below the main carina and along its medial wall and continuing down to approximately 1 cm above the intermediate carina separating the take-offs to the left upper and lower lobes. The injury itself included a portion of bronchial mucosa emanating into the airway although the airway was fully patent. There was no evidence of residual injury, or leak and distal airways were fully patent (Figure 2). A single blood clot was evacuated from the airway. 

The patient was allowed to emerge from anesthesia in the postanesthesia care unit and was making excellent respiratory effort with adequate tidal volumes. She was successfully extubated and observed for any signs of respiratory difficulty in the PACU for approximately 2 hours. She was discharged to the ICU for observation and recovered without any further complication.

## 3. Discussion

 In 1949, the Carlens double lumen endotracheal tube [4] was developed and successfully used to separate lungs and facilitate single lung ventilation. Since that time, DLTs have been widely used for isolated lung ventilation in thoracic and cardiac surgeries as well as in cases of single lung trauma or infection. Placement of these endotracheal tubes is relatively simple in experienced hands and rarely is associated with complications. However, because of the large external tube diameter and the stiff stylette, placement of a DLT is not completely without risk.

 Tracheobronchial rupture during DLT placement is a potentially life-threatening complication but occurs very rarely, likely less than 0.2% of DLT intubations [5]. In fact, between 1998 and 2010, only six reported cases of tracheobronchial injury were published [6–8]. Most commonly, injuries were associated with the distal trachea and/or left main bronchus, likely because of the strong preference to place left sided DLTs. These injuries usually occur as a longitudinal tear within the membranous portion of the trachea. Though tracheobronchial rupture incidence is very low, possible risk factors for such an occurrence might include placement by an inexperienced airway manager, multiple intubation attempts, placement of the stylette through the DLT in such a way that the tip emerges from the luminal tip, overinflation of the cuff, too large of a tube size, incompletely anesthetized patient, weakened membranous trachea secondary to chemotherapy, steroid use or radiation therapy, and previous tracheomalacia. 

When a tracheobronchial tear does occur, it can result in life-threatening complications. Symptoms of bronchial rupture recognized during a surgical procedure can include tension pneumothorax or subcutaneous emphysema. If unrecognized during surgery, this type of tear can result in thoracic cavity infection or sepsis. Early recognition of tracheobronchial tear and prompt repair are essential for optimal patient outcome. Anesthesia provider recognition might include abrupt changes in vital signs, difficulty with ventilation, and the onset of a significant and unexplainable ventilatory leak. 

In the case presented here, the tear was not initially recognized presumably because of ongoing successful ventilation of the injured lung due to correct placement of the inflated bronchial balloon below the distal portion of the tear. In the absence of ventilation difficulty or vital sign changes, we did not suspect any problem to have occurred. A large air leak following deflation of the bronchial cuff manifested by persistent air bubbles in the surgical field alerted the surgeon of a potential airway disruption. Concurrently, we detected a large leak in our ventilatory circuit. Prompt identification of the bronchial tear and proper patching of the defect prevented further sequela from the injury. In addition, inspection of the bronchus by an experienced bronchoscopist was necessary to exclude the possibility of a distal tear that was not observed in the initial surgical repair. As in most cases of recognized tracheobronchial tears caused by DLT placement, our patient experienced a favorable outcome.

In conclusion, a tracheal rupture after intubation with a double lumen endotracheal tube during an operation is very rare. Prompt recognition and repair are important to optimal patient outcome and prevention of further endobronchial as well as systemic complications. Immediate inspection of the airway by an experienced bronchoscopist is necessary to ensure that proper repair has occurred and no further airway injury is present. Finally, prompt extubation of the patient is preferred to avoid further injury by either positive pressure ventilation or movement of the endotracheal tube causing disruption of the repair.
